# Shared dysphoric experiences activate identity fusion, but not forever

**DOI:** 10.1111/bjso.70026

**Published:** 2025-12-14

**Authors:** Hend Bautista, Sara Fregenal, Alexandra Vázquez, Ángel Gómez, Mercedes Victoria Martínez Díaz

**Affiliations:** ^1^ Departamento de Psicología Social y de Las Organizaciones Universidad Nacional de Educación a Distancia Madrid Spain; ^2^ ARTIS International St. Michaels Maryland USA; ^3^ Facultad de Ciencias de la Salud Universidad Rey Juan Carlos Alcorcón Spain

**Keywords:** causes of fusion, identity fusion, shared dysphoric experiences, willingness to fight and die

## Abstract

Identity fusion is a synergistic union of the personal self and target of fusion that predicts extreme behaviours on its behalf. Previous work identified that intense shared dysphoric experiences cause fusion with groups, but no research to date has investigated changes in fusion before, during and after a collective traumatic experience. Six repeated cross‐sectional surveys conducted in Spain (2017–2022) (Study 1) showed that differences between sample means in fusion with the country increased during the COVID‐19 pandemic but decreased when the COVID‐19 subsided. In addition, an experiment provided support for these results (Study 2), since making salient the COVID‐19 crisis (vs. neutral situation) increased fusion with the country. Finally, three additional repeated cross‐sectional surveys conducted in Ukraine (Study 3), one month before the war, one month after the war began and eight months later, replicated that differences between sample means in fusion increased just when the war started, but decreased when the conflict turned chronic. The effects were replicated for fusion with a value, democracy. Apparently, dysphoric experiences represent a temporary drive of fusion, but not a maintenance factor.

## INTRODUCTION

Identity fusion involves a synergistic union of the personal self with a target of fusion (e.g., a group, value or leader), wherein individuals channel their personal agency into extreme behaviours on behalf of the target (Gómez et al., 2020; Swann et al., 2012, 2024). Since its emergence, fusion research has primarily focused on studying its possible consequences and identifying mediators and moderators of these outcomes (see Gómez, Vázquez, Blanco, & Chinchilla, 2024 for a review). Paradoxically, less effort has been made to identify what causes fusion. One possible reason for this oversight is that fusion has been traditionally considered irrevocable (Swann et al., 2012). However, recent studies (Gómez et al., 2019; Kapitány et al., 2019; Lobato & Sainz, 2020; Misch et al., 2018; Misiak et al., 2025; Vázquez et al., 2017) suggest that this irrevocability cannot be taken literally. As Swann et al. (2024, p. 13) pointed out, ‘apparently, fusion may be resilient, but it is not irrevocable’.

Although, three sources have been identified as generators of fusion—being verified by group members (Gómez, Vázquez, Alba, et al., 2024; Rousis et al., 2023), perceiving a shared biological or psychological essence (Swann et al., 2014) and sharing emotional experiences with fellow members (Reese & Whitehouse, 2021)— the most theoretically developed have been *shared experiences*. Living shared experiences means that events such as painful rituals or the horrors of frontline combat can trigger negative affects that promote fusion with the associated group (Whitehouse et al., 2017; Whitehouse & Lanman, 2014). This evidence has been shown in various contexts (i.e., natural disasters: Segal et al., 2018; see also Henríquez et al., 2022; Henríquez & Urzúa, 2023) or populations (i.e., Libyan insurgents, Muslim fundamentalists and Brazilian football hooligans; Whitehouse, 2018). Despite being the most extensively researched line, the long‐term persistence of the effects of these shared experiences on identity fusion has not been thoroughly studied.

As mentioned, the original identity fusion theory, and the recent comprehensive identity fusion theory (Swann et al., 2012, 2024, respectively), point out that shared dysphoric experiences may trigger fusion with fellow group members (Newson et al., 2018, 2021; Whitehouse, 2018; Whitehouse et al., 2014, 2017). However, much of the current research is either cross‐sectional (i.e., Gómez et al., 2019; Vázquez et al., 2017) or focuses on temporally proximate moments (i.e., six days: Misch et al., 2018; three months: Lobato & Sainz, 2020; seven weeks: Kapitány et al., 2019; or thirteen weeks: Zabala et al., 2024), which limits our understanding of fusion's long‐term maintenance (see Misiak et al., 2025 for an exception, who analysed the effects of imagistic events on fusion during nine months). Our work focuses on shared experiences, specifically considering the question of temporality. Therefore, the present research analyses the emergence and long‐term evolution of fusion with a group (i.e., country) in response to two current and dramatic shared negative events: the COVID‐19 pandemic crisis in Spain and the war in Ukraine within people living in the country/Ukrainians. Additionally, while previous research has examined the potential antecedents of fusion with groups and with values (Buhrmester et al., 2018; Reinhardt & Whitehouse, 2024, 2025a, 2025b), here, a shared dysphoric experience in a real context—the war in Ukraine—was explored as a cause of fusion with a value such as democracy.

To understand why it is fundamental to determine the factors that cause and/or increase identity fusion, a brief introduction to identity fusion theory and its consequences is provided.

### Identity fusion, nature and consequences

Identity fusion is defined as a synergistic and visceral feeling of unity with a group, individual, value or abstraction, fostering a deeply ingrained and almost irreversible sense of personal agency and responsibility toward the fusion target (Swann et al., 2024). Although initially conceptualized as a form of alignment between the personal self (e.g., personal identity) and the social self (i.e., the group to which the individual belongs) (Gómez, Brooks, et al., 2011; Swann et al., 2009), recent theoretical developments (Swann et al., 2024) have broadened the concept to encompass diverse targets, such as religious groups (Fredman et al., 2017), abstractions (e.g., gun rights [Martel et al., 2021]; Cecil the Lion event [Buhrmester et al., 2018]), values (e.g., freedom, democracy, honour [Gómez et al., 2022, 2023]) or shared suffering in the Israeli‐Palestinian context and humanity and climate action (Reinhardt & Whitehouse, 2024; Reinhardt & Whitehouse, 2025a; Reinhardt & Whitehouse, 2025b). The inclusion of immaterial targets (e.g., democracy) in identity fusion research has proven particularly valuable due to their strong predictive power for extreme behaviours. Strongly fused individuals perceive themselves and the fusion target as mutually reinforcing, fostering a sense of invulnerability. For these individuals, both personal identity and the identity associated with the fusion target remain simultaneously activated, which accounts for the synergistic nature of fusion. This dual activation strengthens both the sense of personal agency and the feeling of oneness characteristic of identity fusion (Gómez, Brooks, et al., 2011).

Although identity fusion theory is mainly focused on intragroup processes (Gómez et al., 2020; Gómez, Brooks, et al., 2011; Gómez, Morales, et al., 2011; Swann et al., 2009, 2012), it has been extensively studied as a robust predictor of willingness to engage in conflict (Whitehouse et al., 2014), demonstrating its capacity to forecast extreme group behaviours (Varmann et al., 2023) in both laboratory and field studies. For instance, experimentally increasing arousal through physical exercise (i.e., run wind sprints or play dodgeball) triggered the tendency in strongly fused people to increase pro‐group behaviours such as donating personal funds to a needy group member or enacting pro‐group motor activity (i.e., racing a group‐related avatar) (Swann, Gómez, Huici, et al., 2010). Similar effects have been tested on other actions such as the willingness to fight and die for the group (Gómez et al., 2009; Gómez, Brooks, et al., 2011; Gómez, Morales, et al., 2011; Swann et al., 2009), self‐sacrifice in scenarios like the trolley dilemma (Swann, Gómez, Dovidio, et al., 2010) or writing letters of support in the Bombing Marathon of Boston (Buhrmester et al., 2015). Additionally, field research has provided support to those controlled situations, with examples including Israeli acts of retaliation during the 2015 Palestinian intifada (Fredman et al., 2017), inter‐tribal warfare in Cameroon (Buhrmester et al., 2022) or endorsement of honour violence, including behaviours such as slapping or disowning one's daughter (Ashokkumar & Swann, 2023). Moreover, field investigations conducted in Spanish prisons indicated that imprisoned former terrorists who strongly fused with their religion exhibit a greater willingness to make costly sacrifices for their values, in this case their religion compared to fellow terrorists who reported weaker levels of fusion (Gómez et al., 2021, 2022, 2023).

Research examining the consequences of fusion is relevant; however, identifying its causes becomes essential, particularly in light of the outcomes associated with fusion with malevolent groups. The next section addresses the potential causes of identity fusion.

### Shared experiences as causes of identity fusion

Sharing emotionally intense and transformative experiences among individuals belonging to the group has been shown to be a significant factor contributing to the emergence of fusion. Research suggests that identity fusion often arises from shared emotional experiences (see Reese & Whitehouse, 2021). Those experiences, particularly the negative, traumatic or dysphoric ones, tend to foster greater fusion with the group (Kapitány et al., 2019; Misch et al., 2018; Newson et al., 2018). For instance, painful initiation rituals of some groups or the extreme experiences shared by frontline combatants, can induce negative affect that fosters a strong fusion with the associated group (Whitehouse et al., 2017; Whitehouse & Lanman, 2014). Interestingly, the connection between shared experiences and fusion with the group has been demonstrated in different contexts and populations, including Libyan insurgents, Muslim fundamentalists in Indonesia and Brazilian football hooligans (Whitehouse, 2018). Conversely, engaging in positive collective activities, such as folkloric marches or religious celebrations enhances identity fusion, through promoting emotionally charged shared experiences that foster interpersonal and group connections (Páez et al., 2015; Zumeta et al., 2016). These events often generate feelings of emotional synchrony and evoke *kama muta*, a positive relational emotion, which recent research has shown to play a key role in strengthening and maintaining identity fusion (Zabala et al., 2024). Specifically, rituals like collective clapping during the COVID‐19 pandemic were associated with higher perceived emotional synchrony, which in turn predicted greater moral obligation to social norms related to public health measures (Zlobina & Dávila, 2022).

Nevertheless, a limitation of studies on fusion and shared experiences is that most of them are primarily cross‐sectional and correlational (cf. Newson et al., 2018; Vázquez et al., 2017). Only two research studies to date have experimentally manipulated dysphoric experiences. In the first one, the impact of the 2013 marathon attacks was examined to determine whether its salience increased participants' fusion feelings (Jong et al., 2015). Participants who recalled the incident as highly negative reported higher identity fusion than those who remembered it less negatively. In the same vein, those who recalled the shared experience of a natural disaster—in this case, the 2011 Christchurch earthquake— and felt more fear, increased fusion with their community independently of the personal harm they suffered (Segal et al., 2018).

Furthermore, only the medium‐term effect of shared experiences on fusion has been tested. For example, experiences of the massive and collective ritual *Korrika* (a large‐scale relay race across the Basque Country that is both festive and highly symbolic, organized to promote and reassert the Basque language) were measured at three moments: three weeks before, immediately after participation and seven weeks afterwards (Zabala et al., 2024). Similarly, experiences just after completing the Camino de Santiago pilgrimage, at the finishing point and three months later when recalling episodic memories of the pilgrimage through contact with other pilgrims have been examined (Lobato & Sainz, 2020). Political changes and their effect on fusion have also been measured at different time points; for instance, President Trump's Inauguration was measured during a 7‐week period (Kapitány et al., 2019). Similarly, the impact of the event shock of losing or winning the elections was analysed by measuring identity fusion before and after the voting (Misch et al., 2018). And, in the United Kingdom, identity fusion was assessed before and after Brexit and the lockdown (Misiak et al., 2025). In all those pieces of research, the results showed that identity fusion remained high despite the time lapse. However, although previous research has examined fusion in enduring or chronic contexts (e.g., civil conflict, intergroup trauma), the long‐term effects of such experiences have rarely been investigated using multi‐wave designs that capture changes over time.

To fill this gap, the association between dysphoric experiences and increases in identity fusion will first be examined. This will be followed by a test of the effect using a causal approach. Specifically, two recent extreme experiences—the COVID‐19 pandemic and the Ukrainian war—will be analysed to assess whether the increase in fusion is sustained or returns to baseline levels when the experience diminishes (COVID‐19) or becomes chronic (war). Therefore, the aim of this research is to explain changes in identity fusion at the population level through shared dysphoric emotional experiences, specifically the COVID‐19 pandemic in Spain and the war in Ukraine.

## OVERVIEW

The aim was addressed across three studies using two distinct methodological approaches: repeated cross‐sectional (Studies 1 and 3) and experimental (Study 2). Study 1 and Study 2 were conducted online in Spain (using the snowball technique, such that undergraduate students in a distance learning university asked their acquaintances to participate in online research about intergroup relations). Study 3 was conducted online in Ukraine (in coordination with ARTIS^1^ International personnel on the ground in Ukraine).

It was hypothesized that fusion with the country would be higher when an intense shared dysphoric experience emerged (the COVID‐19 crisis for Study 1 and the war in Ukraine for Study 3). However, once the dysphoric experience subsided (the COVID‐19 crisis) or turned long‐lasting (the war in Ukraine), the level of fusion would be lower. This hypothesis was experimentally tested by making salient the threat of the COVID‐19 pandemic (Study 2). Finally, it was also anticipated that the increase and decrease of fusion when the dysphoric experience emerged and turned almost ineradicable would be extended to fusion with values, in this case, democracy (Study 3).

Note that in Study 1, each wave included different participants, and the data were collected at different time points, but for the statistical analyses, we considered the year of the data collection as the independent variable to compare whether the level of fusion among the general population varied in time. The method and protocol for sample collection remained consistent in Study 3.

## STUDY 1. THE COVID‐19 PANDEMIC INCREASED FUSION WITH THE COUNTRY, WHICH DECREASED WHEN COVID‐19 SUBSIDED

Six online repeated cross‐sectional surveys were conducted between 2017 and 2022 to test whether differences in sample means of fusion with the country varied over time. A post‐hoc analysis was conducted to examine whether these differences in fusion were higher when the pandemic emerged, and whether they were lower as the pandemic subsided.^2^

### Method

#### Transparency and openness

Although the studies' design, and its analyses were not pre‐registered, for each study, we report all data exclusions (if any), all manipulations and all measures. Data were analysed using SPSS, version 27.0. All data, materials and analysis code have been made publicly available at OSF (Fregenal, 2025). Approval for Study 1 and Study 2 was obtained from the university of the first author (UNED Human Subjects Approvals) and from ARTIS International (IRB 00007516) for Study 3.

#### Participants

A non‐representative sample of 22,826 individuals participated in Study 1. All participants in these studies were Spaniards, 61.7% women and 38.3% men. A sensitivity analysis using G*Power (Erdfelder et al., 1996) indicated that an effect of *f* = .02 (*η* ^2^ = .002) with 80% power in a one‐way ANOVA (fixed effects, omnibus, one‐way) could be detected for each wave in Study 1.

Although the survey was initially distributed among university students, the resulting sample can be described as a community sample rather than a representative population sample. This distinction is important, as data were collected through snowball sampling initiated from student networks. Nevertheless, the sample was diverse and suitable for the present analyses for two main reasons. Firstly, participants often shared the survey link with family members and friends (typically between four and seven individuals), resulting in responses from a wide range of individuals across different age groups. Secondly, the university offers distance education programmes that attracted a considerable number of adult learners from across the country, further contributing to the demographic diversity of the sample. This pattern is consistent with previous evidence showing positive attitudes toward science and technology among these learners (Gómez Garrido et al., 2006). Therefore, while the findings should not be interpreted as reflecting general population trends, they can be considered indicative of community‐level patterns of the studied variables. Sociodemographic variables are described in Table 1 in the Supporting Information.

#### Procedure

The procedure was the same through all studies: participants were recruited using a snowball procedure and completed the questionnaire by using Qualtrics. All studies were opened for a month (November of each year) and then closed. Participants received a link and answered the questionnaire online, typically from their homes.

After reading the information sheet and informed consent, participants completed the 7‐item scale of the verbal measure of *identity fusion* with the country from Gómez, Brooks, et al. (2011), ranging from 0 (= *totally disagree*) to 6 (= *totally agree*), where higher scores reflected higher fusion with the country. Example items are ‘I am one with my country’, ‘I am strong because of my country’ and ‘I make my country strong’. Alphas = .88, .89, .88, .88, .88 and .88 for each wave, respectively. Finally, participants were debriefed and thanked.

### Results

**FIGURE 1:**
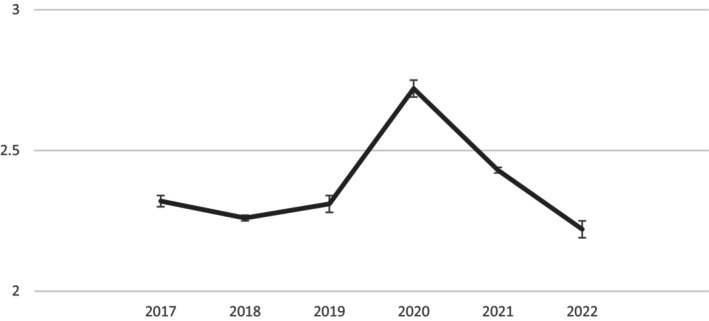
Study 1. Means of identity fusion with the country from 2017 to 2022. The 95% confidence intervals for each year are as follows: 2017, 95% CI [2.27, 2.36]; 2018, 95% CI [2.23, 2.29]; 2019, 95% CI [2.24, 2.37]; 2020, 95% CI [2.66, 2.78]; 2021, 95% CI [2.39, 2.46]; 2022, 95% CI [2.14, 2.29]. Error bars represent ±1 *SE*.

A one‐way ANOVA including the year of the data collection as the predictor showed significant differences in fusion *F*(5, 22803) = 42.69, *p* < .001, $\eta_{p}^{2}$ = .009 (see Figure 1 and Table 1 including means and standard deviations).

**TABLE 1:** Means and standard deviation of identity fusion with the country from 2017 to 2020. Comparisons between years.

Years		*M* (*SD*)	*F*	*t*	*df*	95% CI	*d* Cohen
2017	2017	2.32 (1.38)					
	2018	2.26 (1.39)	1.37	1.82	11,816	−0.003, 0.08	.04
	2019	2.31 (1.42)	2.88	0.19	5190	−0.05, 0.06	.01
	2020	2.72 (1.49)	14.97***	−10.18	5433	−0.33, −0.23	.27
	2021	2.43 (1.40)	0.38**	−3.72	9022	−0.12, −0.04	.07
	2022	2.21 (1.35)	2.09	2.19	4459	0.01, 0.14	.08
2018	2019	2.31 (1.42)	0.99	−1.25	10,456	−0.08, 0.02	.03
	2020	2.72 (1.49)	12.73***	−13.34	10,699	−0.37, −0.27	.31
	2021	2.43 (1.40)	0.36***	−6.94	14,288	−0.15, −0.08	.12
	2022	2.21 (1.35)	5.62	1.16	9725	−0.02, 0.09	.03
2019	2020	2.72 (1.49)	3.51**	−8.96	4073	−0.34, −0.22	.28
	2021	2.43 (1.40)	1.72*	−3.27	7662	−0.14, −0.04	.08
	2022	2.21 (1.35)	6.92	1.83	3099	−0.01, 0.14	.07
2020	2021	2.43 (1.40)	13.73***	8.03	7905	0.15, 0.25	.20
	2022	2.21 (1.35)	17.55***	9.66	3342	0.27, 0.42	.35
2021	2022	2.21 (1.35)	3.74***	4.85	6931	0.09, 0.22	.15

*Note*: ***p < .001, **p < .01, *p < .05.

As Table 1 shows, fusion with the country was significantly higher in 2020—the year of the pandemic—as compared to 2017, 2018, 2019, 2021 and 2022. Also 2021 was significantly higher than 2017, 2018, 2019, 2020 and 2022. Once the COVID‐19 crisis subsides, fusion with the country returned to baseline levels.

### Discussion

Our findings indicate that the differences between sample means in fusion with the country were stable between 2017 and 2019, during the pre‐COVID‐19 period, consistent with the relative stability of identity fusion and its tendency to be resilient (Swann et al., 2024). Moreover, in line with previous findings indicating that dysphoric experiences shared with ingroup members increase fusion with the country (Jong et al., 2015; Kapitány et al., 2019; Newson et al., 2021; Segal et al., 2018; Whitehouse et al., 2017), our results point out that differences between sample means in fusion with the country were significantly higher in 2020, when COVID‐19 emerged, as compared to each of the three previous years. However, such differences in fusion started to decrease one year after COVID‐19, in 2021, but maintained levels significantly higher than the pre‐COVID period. Once the restrictions fully disappeared in 2022, differences between samples on fusion levels with the country decreased and returned to their pre‐COVID‐19 levels.

To the best of our knowledge, this is the first investigation that empirically demonstrates that the level of fusion with the country was higher while shared dysphoric experiences occurred, but that once that experience was over, the levels of fusion returned to their baseline. Although these results are highly valuable, they are not without limitations. Primarily, the methodology, as it is a correlational study, presents two issues that cannot be addressed. First, since different samples are used each year, it is not possible to control for within‐subject variability through randomization of the sample. More importantly, causality cannot be established. While it is true that fusion levels significantly increased during the most challenging years of the COVID‐19 pandemic, other factors, such as political management (Reicher & Stott, 2020b), may have contributed to this increase. To address this limitation, a follow‐up experimental study was designed, highlighting the most challenging moments of the pandemic.

## STUDY 2. THINKING ON THE COVID‐19 CRISIS INCREASES FUSION WITH THE COUNTRY

A follow‐up study tested whether making salient an intense shared dysphoric experience like the COVID‐19 pandemic increased fusion with the country, as compared to a no activation condition. The target of the study provides empirical support for the previous set of studies. It should be noted that the COVID‐19 pandemic was an event, which had direct repercussions (i.e., adherence or resistance) on the attitudes and behaviours of the population related to the types of government policies (Reicher & Stott, 2020a, 2020b). Spain was one of the European countries with the highest excess deaths and the toughest restrictions (Andrino et al., 2021). Specifically, the first lockdown was decreed by the government on 14 March 2020, and lasted several months. It included closure of businesses and public spaces, social distancing rules and travel restrictions, among others. After a reopening phase in spring–summer 2020, new restrictions and confinements were decreed in October until May 2021, just at this moment, the study was carried out. The negative consequences of the pandemic and restriction measures in Spanish society have been consistently proven (Abril et al., 2021; Lasa et al., 2020; Sandín et al., 2020).

### Method

#### Participants

Four hundred and twenty‐three undergraduate students (66.9% women, mean age = 37.79, *SD* = 13.32, 100% Spaniards) volunteered for course credits. No participants were excluded from the analyses. A sensitivity analysis using G*Power (Erdfelder et al., 1996) indicated that a sample size of 423 participants could detect an effect of *f* = .14 (*η* ^2^ = .02) with 80% power in a one‐way ANOVA (fixed effects, omnibus, one‐way).

#### Procedure

Participants were invited to complete an online questionnaire about group processes via Qualtrics. After reading the information sheet and informed consent, participants were randomly assigned either to the experimental condition (*n* = 216, 63.4% women, mean age = 37.32, *SD* = 13.75, 100% Spaniards) or to the control condition (*n* = 207, 70.5% women, mean age = 38.29, *SD* = 13.75, 100% Spaniards). Participants in the experimental condition were asked to remember the toughest months of the COVID‐19 pandemic and to describe how they felt during that time. Examples of participants' responses were: ‘I felt very helpless, and the situation was causing me a lot of anxiety’, ‘I felt constant fear for myself and my family, especially as I was away from […] family. This caused me even greater uncertainty and fear. Being a high‐risk patient generated even more fear for me, with nightmares and anxiety attacks […]’, ‘I felt trapped and overwhelmed because I couldn't go out when I wanted to’. Participants in the control condition were asked to describe their last trip to their job or school (Vázquez et al., 2023). The analyses of the verbal responses in the experimental and the control conditions indicated that all of them understood the instructions correctly and reported what was asked, so no participants were dropped from the analyses. Right after, identity fusion with the country was assessed by means of the 7‐item verbal scale of *identity fusion* (Gómez, Brooks, et al., 2011; Gómez, Morales, et al., 2011), *α* = .84. Finally, participants were debriefed and thanked.

### Results

A one‐way ANOVA indicated that fusion with the country was higher in the experimental than in the control condition, *F*(1, 421) = 15.14, *p* < .001, *M* = 2.59, *SD* = 1.26 vs. *M* = 2.37, *SD* = 1.23, $\eta_{p}^{2}$ = .035.

Additionally, to better understand the specific content of participants' responses and to examine potential differences in levels of identity fusion across thematic categories, we conducted a qualitative analysis (see Table 2 of Supporting Information). This analysis classified responses into three categories: *intrapersonal* (all those comments that spoke about internal conflicts, emotional struggles or personal reflections were categorized as intrapersonal in nature), *interpersonal* (all those comments that referred to interactions, relationships or dynamics between individuals were categorized as interpersonal in nature) and *other* (disagreements which cannot be resolved and those which do not fit in other categories), allowing us to assess both the nature of participants' concerns and whether references to interpersonal or group‐level issues were associated with higher levels of identity fusion. A one‐way ANOVA indicated that fusion with the country was significantly higher for those participants who wrote about an interpersonal situation (vs. intrapersonal), *F*(1, 210) = 6.31, *p* = .013, *M* = 2.84, *SD* = 1.31 vs. *M* = 2.40, *SD* = 1.19, $\eta_{p}^{2}$ = .029.

### Discussion

As expected, participants who thought about the toughest months of the COVID‐19 pandemic reported significantly higher feelings of fusion with their country than those who thought about a neutral situation. These results replicated previous findings that recalling dysphoric experiences shared with other ingroup members are positively associated with an increase in identity fusion (Jong et al., 2015; Kapitány et al., 2019; Newson et al., 2021; Segal et al., 2018; Whitehouse et al., 2017). Moreover, the qualitative analysis of participants' responses and the comparison of identity fusion levels between categories provided further support for this finding. Results showed that those participants who wrote about an interpersonal situation (vs. intrapersonal) reported significantly higher fusion levels.

Although the results are promising, the studies conducted up to here have three main limitations. Firstly, our previous studies focus on the same dysphoric experience. Then, the fact that fusion increases with the shared experience and later decreases when the dysphoric event subsides might be particular to such experience instead of being extended to other experiences. Secondly, the previous studies, but also earlier research, have indicated the positive association between dysphoric experiences and fusion with groups; it remains to be explored whether such experiences would also have an impact on a cherished value shared with other group members. And thirdly, the small size effect found. Regarding this limitation, we would like to point out that although the effect size was small—as would be expected because identity fusion is resilient (Swann et al., 2024)—such variations in fusion could still have meaningful practical implications, potentially inducing significant changes in related variables.

To overcome the first two limitations, a final set of studies was conducted. To generalize the effects of Study 1 to another context, the focus shifted to the war in Ukraine, and data were collected from Ukrainian citizens living in Ukraine before and several months after the initiation of the war with Russia. In addition, although the initial investigations focused on the consequences of fusion with groups (Gómez, Brooks, et al., 2011; Gómez, Morales, et al., 2011; Swann et al., 2009), the original identity fusion theory explicitly acknowledged the possibility of fusion with other targets such as values (Swann et al., 2012), so to extend the effects of dysphoric experiences on fusion with groups, we also assessed fusion with democracy. In this regard, the relationship between identity fusion and democracy has been tested on several occasions (Atran, 2016; Whitehouse et al., 2014). More recently, research has shown that fusion with political identities can predict extreme behaviours, either in defence of democracy or, paradoxically, to justify authoritarian actions if group identity is perceived as being under threat (Kapitány et al., 2019). Fusion with national values, such as those associated with freedom and democracy, can be a strong predictor of collective extreme behaviours (Gómez et al., 2023).

In our research, democracy was chosen as a focal point because the ongoing war in Ukraine is rooted in events like the Maidan Revolution (or Euromaidan) of 2014. This social movement demanded democratic reforms and political and economic changes to align Ukraine more closely with the European Union and its values, rather than maintaining traditional ties with Russia (Shveda & Park, 2016). This narrative, emphasizing the importance of democracy for Ukraine remains relevant today. Furthermore, President Zelensky has consistently highlighted the threat posed by Russia, warning that it aims not only to undermine democracy in Eastern Europe but also in the United States and other European countries (Dawber, 2023; Gessen, 2024; Schmemann, 2023).

## STUDY 3. THE WAR IN UKRAINE INCREASED FUSION WITH THE COUNTRY AND DEMOCRACY, WHICH DECREASED WHEN THE WAR TURNED CHRONIC

Three repeated cross‐sectional surveys tested whether the differences between sample means in fusion with the country and with democracy were higher at the onset of the war in Ukraine compared to previous levels, and whether such differences were lower when the war became chronic among participants directly experiencing the armed conflict.

### Method

#### Participants

A non‐representative sample of 1479 individuals participated in Study 3. All participants in this study were Ukrainians and residents in different regions of Ukraine.^3^ Sociodemographic variables are described in Table 2. A sensitivity analysis using G*Power (Erdfelder et al., 1996) indicated that an effect of *f* = .07 (*η*s^2^ = .005) with 80% power in a one‐way ANOVA (fixed effects, omnibus, one‐way) could be detected for each wave of Study 3.

**TABLE 2:** Study 3. Age range and proportion of women.

Year	*N*	% years from 18 to 60	% Women/men/other
Before offensive	479	97.0	26.30/64.11/1.87
Initial offensive	574	93.9	32.92/64.63/0.89
After offensive	426	89.0	44.59/49.00/2.42

*Note*: The discrepancy in the percentage of participants in the sex variable is due to those who chose not to respond.

#### Procedure

Three online studies were conducted in Ukraine using the Magi‐Wise survey platform (in coordination with ARTIS International personnel on the ground in Ukraine): shortly before the conflict with Russia began (Before offensive, *n* = 479), during the initial Russian offensive (Initial offensive, *n* = 574) and eight months later during a large‐scale Ukrainian counteroffensive (After offensive, *n* = 426). Participants were asked to indicate their fusion with Ukraine, and with democracy, within a questionnaire that also included questions unrelated to the present research.

*Identity fusion* with both Ukraine and democracy was assessed with the Dynamic Identity Fusion Index (Jiménez et al., 2015). This measure was implemented, in the first place, to prevent participants' fatigue during the study, instead of using the verbal fusion scale (Swann et al., 2009). Furthermore, it has been successfully used in previous research to assess fusion with values (Gómez et al., 2022). It displays a figure made of two circles of different sizes on the computer screen. A small circle to the left represents ‘the self’. A bigger circle on the right represents the group (country) or the value (democracy). Participants were asked to move the small circle until it reached the position that best represented their relationship with the country and with democracy. Higher degrees of overlap of both circles, on a scale from 0 to 1, indicate higher levels of fusion. Finally, participants were debriefed, thanked and dismissed.

### Results

**FIGURE 2:**
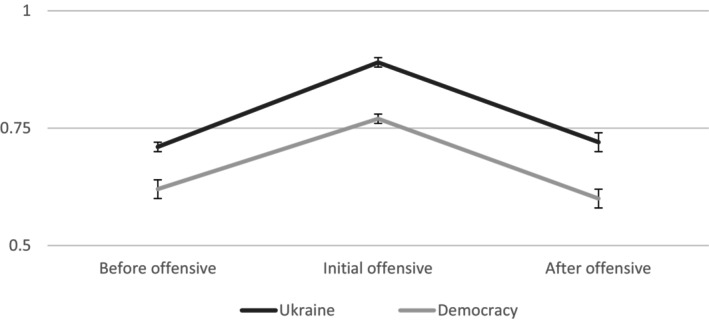
Study 3. Means of identity fusion with Ukraine and with democracy. The 95% confidence intervals for each moment are as follows: Ukraine (before offensive, 95% CI [0.67, 0.75], initial offensive, 95% CI [0.86, 0.91], after offensive, 95% CI [0.68, 0.76]); Democracy (before offensive, 95% CI [0.60, 0.69], initial offensive 95% CI [0.74, 0.80], after offensive 95% CI [0.55, 0.64]). Error bars represent ±1 *SE*.

A one‐way ANOVA including the time for the data collection as the predictor variable showed significant differences between sample means in fusion with the country, *F*(2, 1476) = 34.18, *p* < .001, $\eta_{p}^{2}$ = .044 (see Figure 2 and Table 3 including means and standard deviations).

**TABLE 3:** Means and standard deviation of identity fusion with the country and democracy. Comparisons between before and after the war.

Study		*M* (*SD*)	*F*	*t*	*df*	95% CI	*d* Cohen
Country							
3a	3a	0.71 (0.43)					
	3b	0.89 (0.30)	218.74***	−7.60	1051	−0.21, −0.12	.48
	3c	0.72 (0.43)	0.68	−0.21	903	−0.06, 0.05	.02
3b	3c	0.72 (0.43)	178.00***	7.26	998	0.12, 0.21	.45
Democracy							
3a	3a	0.65 (0.45)					
	3b	0.77 (0.39)	62.74***	−4.69	1051	−0.17, −0.07	.28
	3c	0.60 (0.45)	1.60	1.64	903	−0.009, 0.10	.11
3b	3c	0.60 (0.45)	81.70***	6.39	998	0.11, 0.22	.40

*Note*: 3a pertains to before, 3b to initial and 3c to after offensive. ***p < .001.

As Table 3 shows, fusion with the country was significantly higher during the initial Russian offensive as compared to shortly before the conflict with Russia began. In contrast, fusion with the country was significantly lower eight months later.

Following the same method as in previous studies, a one‐way ANOVA including the time for the data collection as the predictor showed significant differences between sample means in fusion with the democracy, *F*(2, 1476) = 21.55, *p* < .001, $\eta_{p}^{2}$ = .028. As Table 3 shows, fusion with the democracy was significantly higher during the initial Russian offensive as compared to shortly before the conflict and eight months later.

### Discussion

Our findings replicated evidence that differences between sample means in fusion with the country were higher when a dysphoric experience shared with other ingroup members emerged. Additionally, differences between sample means in fusion were lower reaching baseline levels as the dysphoric experience either subsided or turned chronic. For Ukrainian citizens directly living the conflict with Russia the differences between sample means in fusion with the country were significantly higher during the initial Russian offensive as compared to shortly before the conflict with Russia began, but turned back to its original level eight months later, during a large‐scale Ukrainian counteroffensive. Importantly, these results were replicated with a value, democracy.

## GENERAL DISCUSSION

Identity fusion is a psychosocial process characterized by a deep feeling of union between the personal self and a target of fusion (e.g., a group, value or leader). For over ten years, research has consistently shown that fused individuals are willing to engage in extreme behaviours on behalf of the target of fusion (Gómez et al., 2020; Swann et al., 2012, 2024). Traditionally, identity fusion has been considered irrevocable (Swann et al., 2012), and this perception may have led researchers to neglect investigating what could cause or modify fusion and how permanent these changes might be (see Gómez, Vázquez, Blanco, & Chinchilla, 2024 for a review). However, although scarce, recent studies (Gómez et al., 2019; Gómez, Vázquez, Alba, et al., 2024; Lobato & Sainz, 2020; Vázquez et al., 2017; Zabala et al., 2024) show that fusion can be modified and that these changes can be permanent in both the short‐term (Gómez, Vázquez, Alba, et al., 2024) and medium‐term (Lobato & Sainz, 2020; Misiak et al., 2025; Zabala et al., 2024). Nonetheless, to our knowledge, no study addresses fusion over the long‐term, especially when shared experiences become chronic. Therefore, the present research directly addresses this gap by analysing the long‐term changes in identity fusion through two real scenarios that could be considered shared dysphoric experiences: the COVID‐19 pandemic crisis and the war in Ukraine. Additionally, two different fusion targets were considered: the country (Spain and Ukraine) and a value (democracy).

We examined whether identity fusion with groups and with values was higher when intense shared dysphoric experiences occurred. Furthermore, we were interested in knowing whether such an increase is sustained over time. Over three studies we tested whether dysphoric experiences shared with ingroup members, such as the COVID‐19 pandemic or the war in Ukraine increased identity fusion with the country and with democracy. Specifically, the first study, along six waves showed that differences between sample means in fusion with the country were higher when the global COVID‐19 pandemic emerged compared to previous years and were lower when the pandemic subsided, and an experimental study provided causal evidence indicating that recalling the toughest months of the COVID‐19 pandemic fostered greater fusion than recalling a neutral situation. To extend the results to another real‐case scenario, and with another target of fusion, the same measures were administered in a war conflict, Ukraine. Fusion with Ukraine followed the same pattern as with Spain in the context of the COVID‐19 pandemic; that is, it was higher when the war began and returned to its baseline levels when it became chronic. Moreover, this result was extended to a value, democracy.

The present findings replicate previous research supporting those dysphoric experiences cause fusion (Whitehouse et al., 2017; Whitehouse & Lanman, 2014) and they build upon and extend earlier evidence of the association between shared dysphoric experiences and identity fusion. The evidence found up to now evaluated how fusion increased either when a negative event was made salient (i.e., Jong et al., 2015; Segal et al., 2018) or when the relationship between the event and fusion was considered at a certain moment (Kapitány et al., 2019; Misch et al., 2018; Newson et al., 2018). However, the analysis of that change over a long time had not been sufficiently considered until now. Our results show that, although fusion (differences between sample means) was higher when the situation occurred, it was lower and returned to its baseline when that situation became chronic. So far, few studies had directly addressed the analysis of such a temporal dimension, with the longest timeframe considered being nine months (Misiak et al., 2025). In contrast, the current research addressed the temporal dimension by considering a much broader time frame. Variations in fusion responses were evaluated over six years in the case of the COVID‐19 pandemic, and for eight months after the beginning of the war between Ukraine and Russia. On the other hand, changes in fusion levels with democracy have been demonstrated in the context of a shared dysphoric experience, extending the previous results to other targets of fusion as has been recalled in the comprehensive identity fusion theory (Swann et al., 2024) and applying to other scenarios such as world health crises or international wars. Importantly, on a different note, our results were similarly obtained using two different measures of fusion: the verbal scale from Gómez, Brooks, et al. (2011) and the Dynamic Identity Fusion Index from Jiménez et al. (2015), thereby providing convergent validity to our findings.

Furthermore, the results of this research appear to align with previous investigations in other areas related to social identity and collective shared experiences, such as natural disasters. The literature suggests that in times of crisis, the perception of a common fate can lead to the emergence of new groups, fostering pro‐group behaviour, altruism and solidarity (Ntontis et al., 2020, 2022), and even the sense of identity fusion through emotional union with others (Bouchat et al., 2024). However, over time, factors such as perceived lack of social support, inequality or the presence of secondary stressors may contribute to the decline of this sense of unity (Ntontis et al., 2022).

This work is not without limitations. Most importantly, we assume that the increase in fusion is associated with the intense shared dysphoric experiences occurring during both periods. However, other factors could also be responsible (e.g., effective administrative management [Reicher & Stott, 2020a, 2020b], the opportunity to stay at home with family). Nevertheless, it is worth noting that an additional experiment was conducted to enhance the consistency of the results. Control measures should be incorporated to address other potential influences (e.g., affective response, Watson & Clark, 1994; reflection on the event, Cann et al., 2011; or experienced sharedness, Misiak et al., 2025).

Relatedly, we may also be assuming that the COVID‐19 event is inherently negative and traumatic, when in fact it may not necessarily be perceived as such. However, this might not be worth considering if we contemplate an alternative perspective regarding the causes of identity fusion. One of the most well‐documented effects of emotional sharing is its role in strengthening social bonds (for reviews, see Peters et al., 2018; Rimé, 2009). Through this process, individuals not only connect with others, but also reconstruct the meaning of their experience, thereby contributing to posttraumatic growth (Garcia & Rimé, 2019; Rimé, 2020; Tedeschi & Calhoun, 2004). This emotional sharing could explain, for instance, why during painful rituals, positive affect arises (Fischer et al., 2014; Kavanagh et al., 2019), suggesting that independently of the positive or negative valence of the experience, what really matters is the perception of it being essential and shared with others (Zabala et al., 2024). Events like natural disasters or attacks can give rise to collective rituals such as demonstrations (Gasparre et al., 2010; Páez et al., 2005, 2013; Pelletier, 2018; Rimé et al., 2010) or, in the case of the pandemic, to rituals like collective clapping (Zlobina & Dávila, 2022). Therefore, it may be that, in this study, individuals might have experienced the event either negatively or positively—but most importantly, as shared or reinterpreted it as shared through the process of emotional sharing. This sense of unity or group consciousness can emerge through both top‐down and bottom‐up psychological processes (Hobson et al., 2018). Perhaps the interpretation of our findings calls for an explanation rooted primarily in top‐down processes namely, collective emotions or emotional convergence among individuals facing a shared situation (von Scheve & Ismer, 2013).

Secondly, it is important to carefully consider the identity fusion levels reported by participants, especially in Study 1, where the highest average score was 2.72 on a 7‐item scale ranging from 0 to 6. While these statistical analyses reflect meaningful changes in fusion (e.g., moderate effect sizes are significant in this field), these averages may indicate a weak group connection rather than authentic fusion as theorized. This raises the question of whether identity fusion is ‘revocable’ or ‘resilient’, as suggested by the comprehensive identity fusion theory (Swann et al., 2024). Nevertheless, we may also need to acknowledge that, in Spain, fusion levels are never too high (e.g., Swann et al., 2014 for a comparison between countries), suggesting that cultural factors might also play a role. This ambiguity limits conclusions and highlights the need for more sensitive (i.e., pictorial scale of identity fusion; Swann et al., 2009) or complementary measures (i.e., identification; Mael & Ashforth, 1992) to capture explicitly changes in fusion levels. Accordingly, the present findings should be interpreted as consistent with identity fusion theory, rather than as providing direct evidence for mechanisms specific to fusion. Third, there is room for improvement in the research design. Although the repeated cross‐sectional design allows us to have many participants as well as observe variations over time, it has the limitation that these variations are not within subjects (i.e., it is not a longitudinal design), but within populations, so the results must be interpreted cautiously. Nonetheless, when interpreted with caution, our findings offer valuable insights as they incorporate certain recommendations pertinent to this kind of investigation (i.e., adequate sample representation, sufficient temporal variability, consistency in measurement and considering the context of application; Johnston & Brady, 2002). Alternatively, the experimental manipulation in Study 2 may not have been exactly comparable, and thus the observed differences may not be solely attributable to emotional valence, the shared nature of the experience or both. Future research should therefore include a neutral valence but shared condition to disentangle these effects. Additionally, although valuable and reliable, our results show evidence for the target of fusion being evaluated (i.e., country and democracy) and thus cannot be generalized to other groups or values. Further research is needed to explore additional factors contributing to fusion with other targets.

Regarding above, although most research on identity fusion has focused on fusion with the country (Varmann et al., 2023 for a review), the recently comprehensive identity fusion theory (Swann et al., 2024) highlights that people may feel fused with targets other than the country. Entities such as romantic partners (Joo & Park, 2017; Walsh & Neff, 2018), leaders like Donald Trump (Kunst et al., 2019), brands (Krishna & Kim, 2020, 2021; Lin & Sung, 2014) or animals (Buhrmester et al., 2018) have been contemplated in the literature about fusion. For instance, imprisoned individuals convicted of terrorism‐related offenses reported feeling fused with their religion (value), which was positively associated with a greater propensity to make costly sacrifices in its name (Gómez et al., 2021). Similarly, a value like freedom has also been evaluated among Spanish participants in relation to the war in Ukraine. Consistent with previous results, fusion with freedom predicted the willingness to fight for both Ukraine and freedom (Gómez et al., 2023). The entity with which individuals feel fused is very important when assessing what kind of sacrifices they will be willing to make (Gómez et al., 2022). This can be crucial in situations where people feel fused simultaneously with their groups and with values, they share with them (Gómez et al., 2017). Therefore, conducting research that covers other fusion entities to consider different contexts, realities and sacrifices is essential.

Another limitation of this study is related to the measurements and the control of experimental manipulation. In the first place, some identification measure (Mael & Ashforth, 1992) could have been included to isolate the effect of intense shared experiences on identity fusion.

In a different vein, while we measured identity fusion, we did not account for the willingness to sacrifice among fused individuals. Given that fusion is primarily characterized by its predictive nature of extreme pro‐group behaviour (Gómez et al., 2020; Gómez, Brooks, et al., 2011; Gómez, Morales, et al., 2011; Swann et al., 2009, 2012, 2024), determining whether these variations would accompany extreme pro‐target behaviours is crucial. Exploring potential mediators in such scenarios is also essential, as they may vary substantially depending on the contexts. Longitudinal data or experimental manipulation of these mediators would establish the causal relationship of the hypothesized explanatory. Nevertheless, given that the pandemic was unforeseen, conducting a longitudinal study was not feasible. Moreover, it is essential to consider the perceived level of threat arising from both situations, such as the pandemic and the war. Threat has been considered the strongest moderator of the link between fusion and pro‐group violence (Swann et al., 2024) and explicitly incorporated in several fusion‐based theories including the devoted actor model (Atran et al., 2014), the threat‐plus‐fusion model (Whitehouse, 2018) and the fusion‐secure base hypothesis (Klein & Bastian, 2023). Moreover, it is also possible that our manipulation generated rumination instead of the feeling of sharedness. Future research may solve this limitation by providing feedback after recalling the experience. Another option could be assessing the extent to which participants believed that the experience they were describing was shared by other members of their country.

Lastly, it focused exclusively on negative experiences. However, shared positive experiences have also been shown to foster fusion (Zumeta et al., 2020) even stronger than negative ones (Kavanagh et al., 2019). For this reason, and because even in moments like COVID‐19 or war, there are also positive shared experiences, such as applauding (Gómez, 2020), bringing people to safety from the war (Konsevych, 2023) and helping the elderly (Panych, 2024) is required to explore how identity fusion could change when such shared experiences are positive. Despite these limitations, our findings contribute valuably to the study of the nature of identity fusion.

## CONCLUSION

In recent years, the world has faced some of the most intense and negative experiences of the 21st century: the COVID‐19 pandemic and the war in Ukraine. Both contexts meant collectively sharing dysphoric experiences and offered a real scenario to measure the evolution of fusion with the group (country) and value (democracy). Two online repeated cross‐sectional studies showed that identity fusion changes over time, coinciding with the emergence of the COVID‐19 pandemic and the war in Ukraine; however, once the context stabilized, fusion returns to the previous levels. An experiment revealed that highlighting the worst moments of the pandemic increased fusion. These findings suggest that experiencing shared intense and negative events might increase fusion not only with the ingroup but also, with a value. They also indicate that sharing dysphoric experiences can represent a temporary drive of fusion, but not a maintenance factor.

## AUTHOR CONTRIBUTIONS

**Hend Bautista:** Writing – original draft; methodology; formal analysis; conceptualization. **Sara Fregenal:** Conceptualization; supervision; data curation. **Alexandra Vázquez:** Conceptualization; investigation; writing – review and editing; supervision; methodology. **Ángel Gómez:** Conceptualization; investigation; funding acquisition; writing – review and editing; formal analysis; supervision; resources; methodology. **Mercedes Victoria Martínez Díaz:** Conceptualization; investigation; methodology; writing – original draft; formal analysis.

## CONFLICT OF INTEREST STATEMENT

All authors declare no conflict of interest.

## Supporting information


Data S1.

## Data Availability

The data that support the findings of this study are openly available in OSF at https://osf.io/vpmg4/?view_only=e3cc2c8edcf047bdbae9938868449fca.

## Footnotes

^1^
ARTIS is a scientific research organization that focuses on understanding human cognition and behaviour, particularly in contexts of cooperation and conflict. Their work spans behavioural dynamics, conflict resolution and the psychological drivers of decision‐making under stress or extreme circumstances. The organization collaborates with international partners, including universities, governments and non‐governmental organizations, to apply its research to global challenges. Their official website, artisinternational.org, provides detailed information about their projects, publications and initiatives.

^2^
The Identity Fusion Lab systematically conducts annual assessments of identity fusion levels as part of the students' academic work. During these assessments the COVID‐19 pandemic emerged. The nature of the data is cross‐sectional rather than longitudinal, because retaining a sufficiently large and representative sample year after year is particularly difficult in large‐scale surveys.

^3^
They participated online (in coordination with ARTIS, personnel on the ground in Ukraine). Participants' age was measured in ranges. Here we conduct a re‐analysis of the data that appeared at Gómez et al. (2023).
