# Supplementary material for: Shared dysphoric experiences activate identity fusion, but not forever
Source: Br J Soc Psychol. 2025 Dec 14;65(1):e70026. doi: 10.1111/bjso.70026 (PMC12703063; doi:10.1111/bjso.70026)
Supplement: Supplementary file 1 — Data S1. [file BJSO-65-0-s001.docx]

**Supplementary Material**

**“Shared dysphoric experiences increase identity fusion, but not forever”**

**Study 1**

**Table 1**

*Means and standard deviation of age and proportion of women from 2017 to 2022.*

| Year | *N* | *M*_age_ (*SD*) | Min-Max | % Women |
| --- | --- | --- | --- | --- |
| 2017 | 3,275 | 35.95 (12.84) | 18-80 | 62.0 |
| 2018 | 8,535 | 36.70 (12.95) | 18-80 | 61.9 |
| 2019 | 1,909 | 35.59 (13.09) | 18-80 | 64.5 |
| 2020 | 2,157 | 35.21 (13.18) | 18-80 | 60.8 |
| 2021 | 5,748 | 37.06 (14.30) | 18-80 | 60.4 |
| 2022 | 1,185 | 38.25 (14.04) | 18-79 | 62.4 |

**Study 2**

**Qualitative analysis**

To explore and understand the types of responses which participants gave when they spoke about the toughest months of the COVID-19 pandemic and to describe how they felt during that time, a qualitative content analysis of their responses was conducted. Since a specific theoretical framework had already been established to guide the categorization (see introduction, *p*. 7), the researchers developed clear and concrete definitions for each category to ensure mutual exclusivity. Two research assistants, who were not involved in the study’s main objectives, independently analysed the participants’ responses. Therefore, only a deductive analysis of the responses was performed (see Knott et al., 2022). Category assignment was based on the semantic and interpretative content of the data. Each participant’s response was treated as a coding unit and could not be assigned to more than one category, ensuring mutual exclusivity. To refine the categorization process, both coders were allowed to discuss discrepancies in their coding. However, if disagreements could not be resolved, the response was placed in the “Other” category and excluded from further analysis.

***Results***

The thematic analysis revealed three main categories based on participants’ responses provided when asked to describe the toughest months of the COVID-19 pandemic and how they felt during that time. Table 2 presents a summary of the categories, along with illustrative examples for each. Below, we describe each category in detail.

**Table 2**

*Content Analysis Categories, Thematic Indicators, Examples, Inter-Rater Reliability, and Frequencies*

| **Definition of categories** | **Kappa (κ)** | | | *n* (%) |
| --- | --- | --- | --- | --- |
| **Intrapersonal category** | | | | |
| All those comments that spoke about internal conflicts, emotional struggles, or personal reflections were categorized as intrapersonal in nature. | | 1 | | 129 (60.0) |
| “Para mí no fue mal, salía a trabajar y hacía vida medio normal. No tenía miedo.” [Mujer de 36 años] | “It wasn't bad for me. I went to work and lived a semi-normal life. I wasn't afraid.” [36-year-old woman] | | | |
| “Tranquilo y con un poco de incertidumbre” [Hombre de 64] | “Calm and a little uncertain.” [64-year-old man] | | | |
| “Sin control de mi vida. Estresada, frustrada, angustiada” [Mujer de 42 años] | “Without control of my life. Stressed, frustrated, anxious.” [42-year-old woman] | | | |
| **Interpersonal category** | | | | |
| All those comments that referred to interactions, relationships, or dynamics between individuals were categorized as interpersonal in nature. | | 1 | | 83 (38.6) |
| “Pensaba en la gente que se estaba muriendo, sin ver a su familia, sentía la impotencia de no poder hacer nada por los demás, mucho sufrimiento.” [Mujer de 64 años] | “I thought about the people who were dying, without seeing their families. I felt helpless at not being able to do anything for others, so much suffering.” [64-year-old woman] | | | |
| “Me sentía abrumado por la cantidad de muertes y preocupado por la percepción de mis hijos y la mía propia sobre el futuro.” [Hombre de 48 años] | “I felt overwhelmed by the number of deaths and worried about my children's and my own perception of the future.” [48-year-old man] | | | |
| “Los momentos más duros fue cuando sentí esa percepción colectiva de miedo, cuando se salía a la calle y se interaccionaba con la gente (en el mercado, en espacios públicos...). Provocaba mucha tensión interna.” [Mujer de 51 años] | “The toughest moments were when I felt that collective perception of fear, when I went out on the street and interacted with people (at the market, in public spaces, etc.). It caused a lot of internal tension.” [51-year-old woman] | | | |
| **Other category** | | | | |
| Disagreements which cannot be resolved, and those which do not fit in other categories. | | | 1 | 3(1.4) |
|  |  | | |  |
| “Marzo, abril y mayo de 2020” [Mujer de 27 años] | “March, April, and May 2020” [27-year-old woman] | | | |

A one-way ANOVA indicated that fusion with the country was significantly higher for those participants who wrote about an interpersonal situation (vs. intrapersonal), *F*(1, 210) = 6.31, *p* = .013, *M* = 2.84, *SD* = 1.31 vs. *M* = 2.40, *SD* = 1.19, η^2^*_p_*= .029.

**Scales additional information**

**Study 1**

***Fusion Scale with the country***

Participants indicated to what extent they agreed with the following items: “my country is me”, “I am one with my country”, “I feel immersed in my country”, “I feel a deep emotional bond with my country”, “I am strong because of my country”, “I would do for my country more than any other group members would do”, “I make my country strong.” Participants responded using a 7-point Likert scale ranging from 0 (completely disagree) to 6 (completely agree). All items were originally developed in Spanish. A factorial analysis with Oblimin rotation was conducted. All items loaded on a single factor:

| Item | Factor loading |
| --- | --- |
| My country is me | .678 |
| I am one with my country | .783 |
| I feel immersed in my country | .764 |
| I feel a deep emotional bond with my country | .773 |
| I am strong because of my country | .814 |
| I would do for my country more than any other group members would do | .783 |
| I make my country strong | .754 |

**Study 2**

***Fusion Scale with the country***

Participants indicated to what extent they agreed with the following items: “My country is me”, “I am one with my country”, “I feel immersed in my country”, “I feel a deep emotional bond with my country”, “I am strong because of my country”, “I would do for my country more than any other group members would do”, “I make my country strong.” Participants responded using a 7-point Likert scale ranging from 0 (completely disagree) to 6 (completely agree). All items were originally developed in Spanish. A factorial analysis with Oblimin rotation was conducted. All items loaded on a single factor:

| Item | Factor loading |
| --- | --- |
| My country is me | .540 |
| I am one with my country | .795 |
| I feel immersed in my country | .715 |
| I feel a deep emotional bond with my country | .759 |
| I am strong because of my country | .757 |
| I would do for my country more than any other group members would do | .726 |
| I make my country strong | .708 |

**Study 3**

***Dynamic Identity Fusion Index with the country***

Participants had to move the smaller circle, which represented themselves, towards a larger circle, which represented their country, in this case Ukraine, in order to represent how they perceived their relationship with their country. The statement with the instructions was developed in Ukrainian.

**
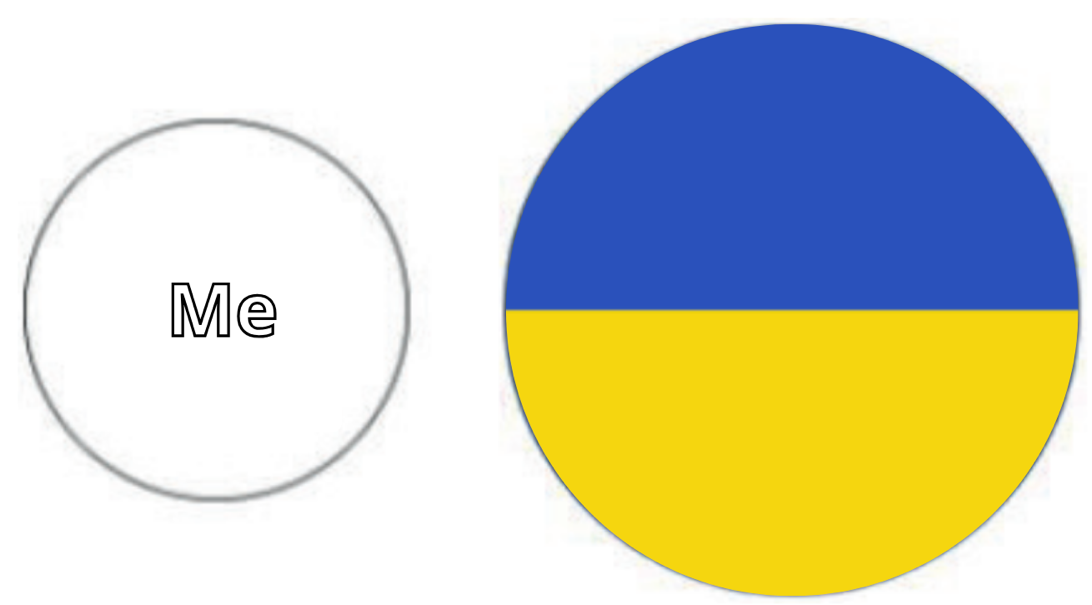
**

***Dynamic Identity Fusion Index with democracy***

Participants had to move the smaller circle, which represented themselves, towards a larger circle, which represented a cherished value, in this case democracy, in order to represent how they perceived their relationship with democracy. The statement with the instructions was developed in Ukrainian.

**
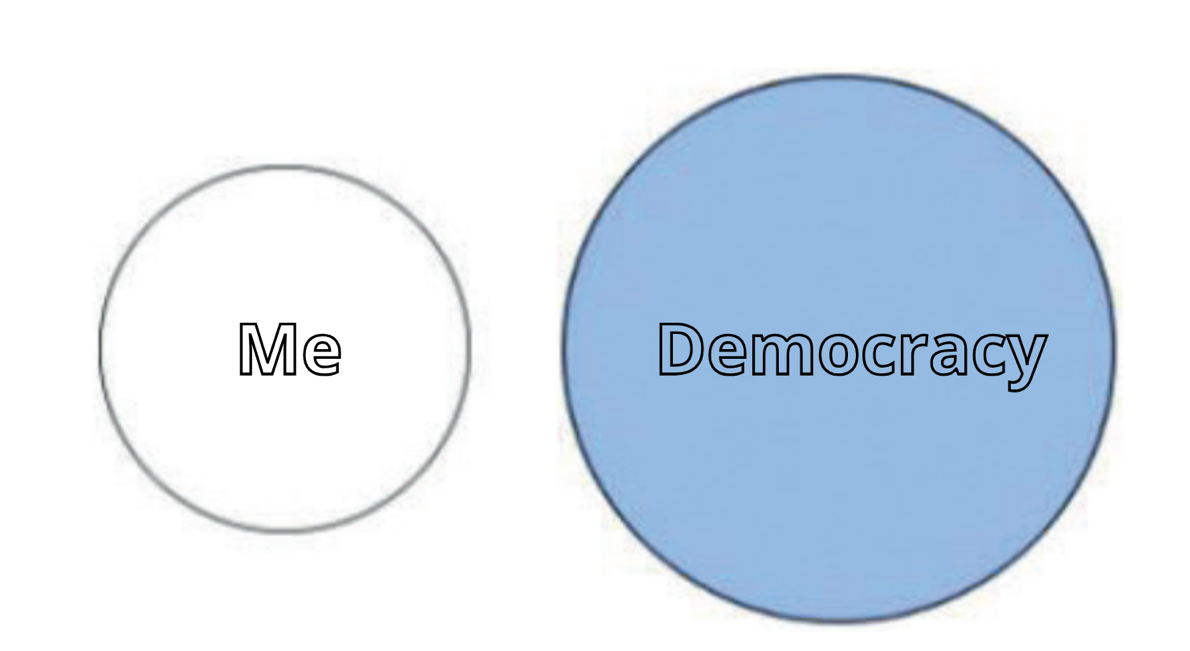
**

**Codebook**

**Studies 1. The COVID-19 Pandemic increased fusion with the country, which decreased when the COVID-19 subsided.**

| Name | Label | Values |
| --- | --- | --- |
| Code |  |  |
| Sex | Sex | 1 = Female, 2 = Male |
| Age | Age |  |
| Nationality | Spanish |  |
| Year |  |  |
| Fusion_1 | My country is me | 0 = Completely disagree to 6 = Completely agree |
| Fusion_2 | I am one with my country | 0 = Completely disagree to 6 = Completely agree |
| Fusion_3 | I feel immersed in my country. | 0 = Completely disagree to 6 = Completely agree |
| Fusion_4 | I feel a deep emotional bond with my country | 0 = Completely disagree to 6 = Completely agree |
| Fusion_5 | I am strong because of my country | 0 = Completely disagree to 6 = Completely agree |
| Fusion_6 | I would do for my country more than any other group members would | 0 = Completely disagree to 6 = Completely agree |
| Fusion_7 | I make my country strong | 0 = Completely disagree to 6 = Completely agree |
| Fusion_mean |  |  |

**Study 2. Thinking on the COVID-19 increases fusion with the country**

| Name | Label | Values |
| --- | --- | --- |
| Code | Participant’s code |  |
| Sex | Sex | 1 = Female, 2 = Male |
| Age | Age |  |
| Nationality | Spanish | 1 |
| Condition |  | 0 = Control, 1 = Experimental |
| Control | Please, explain below how has your last trip to your workplace or to your study centre been | 0 |
| Experimental | Please, think of the toughest moments of the COVID-19 pandemic and describe below how were you feeling in that moment | 1 |
| Fusion_mean | Fusion_mean (Fusion_1 Fusion_2 Fusion_3 Fusion_4 Fusion_5 Fusion_6 Fusion_7) |  |
| Fusion_1 | My country is me | 0 = Completely disagree to 6 = Completely agree |
| Fusion_2 | I am one with my country | 0 = Completely disagree to 6 = Completely agree |
| Fusion_3 | I feel immersed in my country. | 0 = Completely disagree to 6 = Completely agree |
| Fusion_4 | I feel a deep emotional bond with my country | 0 = Completely disagree to 6 = Completely agree |
| Fusion_5 | I am strong because of my country | 0 = Completely disagree to 6 = Completely agree |
| Fusion_6 | I would do for my country more than any other group members would | 0 = Completely disagree to 6 = Completely agree |
| Fusion_7 | I make my country strong | 0 = Completely disagree to 6 = Completely agree |

**Study 3. The war in Ukraine increased fusion with the country and democracy, which decreased when the war turned chronic.**

| Name | Label | Values |
| --- | --- | --- |
| Code | Participant’s code |  |
| Time | Time | 1 = pre-war, 2 = 1 month after war, 3 = 8 months after war |
| Age | Age | 0 = 18-24, 1 = 25-30, 2 = 31-40, 3 = 41-50, 4 = 51 – 60, 5 = 60+ |
| Gender | Gender | 0 = Male, 1 = Female |
| Fus_Democracy | Fusion with democracy. |  |
| Fus_Ukraine | Fusion with Ukraine. |  |
